# MicroRNAs in treatment-induced neuroendocrine differentiation in prostate cancer

**DOI:** 10.20517/cdr.2020.30

**Published:** 2020-10-12

**Authors:** Theresa Akoto, Divya Bhagirath, Sharanjot Saini

**Affiliations:** ^1^Department of Cellular Biology and Anatomy, Augusta University, Augusta, GA 30912, USA.; ^2^Department of Biochemistry and Molecular Biology, Augusta University, Augusta, GA 30912, USA.

**Keywords:** MicroRNAs, neuroendocrine differentiation, castration-resistant prostate cancer, epigenetics, oncomirs, tumor suppressors, androgen deprivation therapy

## Abstract

Prostate cancer is a condition commonly associated with men worldwide. Androgen deprivation therapy remains one of the targeted therapies. However, after some years, there is biochemical recurrence and metastatic progression into castration-resistant prostate cancer (CRPC). CRPC cases are treated with second-line androgen deprivation therapy, after which, these CRPCs transdifferentiate to form neuroendocrine prostate cancer (NEPC), a highly aggressive variant of CRPC. NEPC arises via a reversible transdifferentiation process, known as neuroendocrine differentiation (NED), which is associated with altered expression of lineage markers such as decreased expression of androgen receptor and increased expression of neuroendocrine lineage markers including enolase 2, chromogranin A and synaptophysin. The etiological factors and molecular basis for NED are poorly understood, contributing to a lack of adequate molecular biomarkers for its diagnosis and therapy. Therefore, there is a need to fully understand the underlying molecular basis for this cancer. Recent studies have shown that microRNAs (miRNAs) play a key epigenetic role in driving therapy-induced NED in prostate cancer. In this review, we briefly describe the role of miRNAs in prostate cancer and CRPCs, discuss some key players in NEPCs and elaborate on miRNA dysregulation as a key epigenetic process that accompanies therapy-induced NED in metastatic CRPC. This understanding will contribute to better clinical management of the disease.

## Introduction

Prostate cancer (PCa) remains the second leading cause of cancer death in men after lung cancer, according to the American Cancer Society^[1]^. In 2020, there will be about 191,930 estimated new cases of PCa in men, of which 33,330 are expected to die from the disease^[2]^. The prostate-specific antigen (PSA) test is aimed at detecting PCa at an early stage to monitor disease progression^[3]^. Patients with PCa can be treated by way of active surveillance, radiation and surgery depending on the stage of the tumor^[4,5]^. Androgens stimulate PCa cells to grow since tumor cells express androgen receptor (AR)^[6]^. As a result, androgen deprivation therapy (ADT) is mostly used for treating PCa. After treatment, the disease declines in most patients for some time, but then progresses to castration-resistant prostate cancer (CRPC), which does not respond to hormonal therapy^[7]^. Since AR signaling still drives CRPC, drugs have been designed to either inhibit intratumoral androgen synthesis [e.g., abiraterone (ABI)] or more effectively inhibit AR signaling [e.g., enzalutamide (ENZ)]^[8,9]^. However, this treatment of CRPC ultimately leads to the transdifferentiation of CRPC to neuroendocrine prostate cancer (NEPC), an aggressive subtype of CRPC^[10-12]^. NEPC arises via a reversible transdifferentiation process, known as neuroendocrine differentiation (NED), which is associated with altered expression of lineage markers such as decreased expression of AR and increased expression of neuroendocrine (NE) lineage markers including enolase 2 (ENO2), chromogranin A (CHGA) and synaptophysin (SYP)^[13,14]^. NEPC displays NE cell phenotype and is an AR-negative variant. Since it is AR-negative, these patients show low serum PSA and resistance to AR-targeted therapy^[15,16]^. As a result, there is a need to identify and develop novel therapies for targeting AR-independent mechanisms in advanced PCa. Developing novel therapies require better understanding of the disease. It has been hypothesized that cellular heterogeneity may contribute to the eventual failure of AR-targeted agents^[17]^. MicroRNAs (miRNAs) are small non-coding RNAs that are important gene regulators, acting primarily by suppressing gene expression post-transcriptionally via sequence-specific interactions with the 3’- untranslated regions of cognate mRNA targets^[18,19]^. miRNA dysregulation has been implicated in many cancers^[20]^. This review seeks to address the association of miRNAs with NEPC.

## Prostate gland cell types and emergence of therapy-induced NEPC

**Figure 1:**
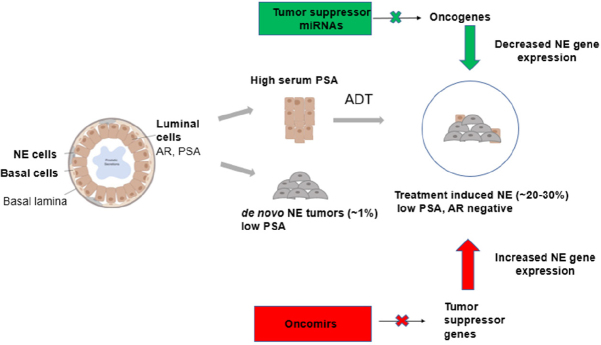
Prostate cancer cell of origin and miRNA regulation. Schematic illustration showing the different cell types of the prostate gland contributing to the formation of CRPC and SCNC and the roles of miRNAs in the regulation of therapy-induced NE. miRNA: MicroRNA; CRPC: castration-resistant prostate cancer; SCNC: small cell neuroendocrine carcinoma; NE: neuroendocrine

The prostate gland is composed of three main cell types (luminal cells, basal cells and NE cells) [Figure 1]. Luminal cells are the most dominant cell type, expressing keratins (K8, K18), AR and PSA^[21]^. Basal cells are the second most dominant cell type and do not express AR and PSA. These cells also express keratins (K14, K5) and stem cell marker p63. NE cells are rare cells interspersed between the basal cells, which express ENO2, CHGA and SYP^[6,22]^. The majority of patients diagnosed with CRPC are characterized by high levels of serum PSA^[23]^. Histologically, prostate tumors are luminal in nature though tumors have also been reported to arise from basal cells, which progress to PCa with luminal-like features^[6,24,25]^. However, after treatment with AR pathway inhibitors such as ENZ and ABI, these luminal cells transdifferentiate to form neuroendocrine cells leading to therapy-induced PCa (NEPC), an aggressive subtype of CRPC characterized by low PSA and no AR signaling^[26-30]^. A small percentage of PCa patients present *de novo* NEPCs that arise directly from NE cells of the prostate gland, and these tumors are independent of treatment as shown in Figure 1^[31]^. Therapy-induced NEPC represents a spectrum of phenotypes, including focal NED in prostate adenocarcinomas, mixed NE/adenocarcinoma or pure NE tumors (small cell/large cell)^[26]^.

### NEPC: molecular features and epigenetics

NEPC is a highly aggressive subtype of PCa^[10]^. It has been shown to progress aggressively, metastasizing primarily to the visceral organs, tissues and sometimes to the bone^[32]^. Histologically, it is characterized by poor differentiation in tumors, coil-like, or organoid shaped cells which appear basophilic with distinct nucleoli^[32]^. In addition, studies have shown that, patients confirmed with NEPC usually develop visceral metastases, have low PSA levels and have frequent loss of retinoblastoma 1 (*Rb1*) and tumor suppressor p53 (*TP53*) genes^[10,33,34]^.

Epigenetic changes are associated with many human cancers particularly with PCa development and progression^[35,36]^. Epigenetics is usually defined as heritable changes in regulating gene expression without changes in the DNA sequence. Histone modifications, DNA methylation, and miRNAs have been shown as epigenetic modifications that play crucial roles in PCa growth and metastasis^[37-40]^. These epigenetic changes have a well-established role in regulating cellular plasticity in NEPC^[41]^. DNA methylation is a key epigenetic modification that affects transcription and development^[42]^. Chromatin flux in NEPC is shown to be regulated by Rb1 and TP53, which can prevent NED^[43]^. Inactivation of both *TP53* and *Rb1* is required to reprogram transcriptional profiles and chromatin accessibility landscapes of normal prostate epithelial cells to human NEPC/small cell prostate cancer. Interestingly, when retinoblastoma protein (pRb) is lost, cells exhibit overexpression of DNA methyltransferase 1 (DNMT1) which leads to an accumulation of DNMT1 protein^[44]^. Studies have shown that DNMT1 is overexpressed in NEPC^[45]^. Furthermore, DNA methylation has been observed to cause downregulation of miR-145, miR-127, miR-124a, and miR-34, suggesting that miRNA epigenetic mechanisms could regulate the tumor epigenome^[46]^. Apart from the loss of *TP53* and *Rb1*, the activation of mitotic programs involving an upregulation of Aurora kinase A (AURKA) and amplification of MYCN have been shown to cause NED due to androgen depletion^[47]^. AURKA and MYCN functionally work together and can induce a NE phenotype in prostate cells, and the overexpression and amplification of MYCN in NEPC tumors and in about 5% of PCas is associated with NED as well as stabilization of AURKA^[10]^. Histone methylation and acetylation are the most common types of histone modifications seen in chromatin which are regulated by enzymes such as: histone acetyltransferases, histone deacetylases, histone methyltransferases (HMTs) and histone demethylases^[48]^. Enhancer of zeste homolog 2 (EZH2) is a HMT that is also overexpressed in NEPC and leads to cellular plasticity during transdifferentiation, and the expression of EZH2 is also regulated directly by miR-101^[49,50]^. In summary, these studies indicate that different epigenetic changes can regulate the epigenome by functioning in concert.

### MicroRNAs: key players in prostate cancer progression

MicroRNAs (miRNAs) are small non-coding endogenous RNA molecules that bind to target mRNAs with complementary bases to degrade it or attenuate translation^[51,52]^. They play a key role in regulating about 60% of mRNAs^[53,54]^. Expression profiles of miRNAs in cell lines and clinical samples have been shown to be unique in a large number of studies and miRNAs have been shown to play an important role in the initiation and progression of many diseases including PCa^[55]^. Therefore, miRNAs have been used as biomarkers and therapeutic targets in diseases^[56]^. In PCa, miRNA levels can be dysregulated by methylation of their promoters or by other tumor-promoting factors or by genomic loss or deletion^[57]^. They can be classified functionally as oncomirs (gain-of-function) or tumor suppressor miRNAs (loss-of-function) based on the roles they play in tumorigenesis^[58,59]^. An important feature of transdifferentiation from an adenocarcinoma state to an NEPC is lack of hormone responsiveness or androgen independence. This is further supported by clinical data from patients where castration-resistant tumors often have focal areas of NE differentiation^[60]^. More studies explore the genetic and epigenetic mechanisms that are activated upon ADTs, eventually leading to the development of NE phenotypes. In a recent study, Zhang *et al*.^[61]^ identified the activation of enhancer of zeste homolog 2 (EZH2)/cAMP response element-binding protein pathway as a result of androgen deprivation promoting NE differentiation in PCa cell line. In the following section, we will review the role of miRNAs that are either directly associated with development of NEPC or may indirectly be associated with NE differentiation due to their dysregulation upon androgen deprivation [Table 1].

**Table 1:** Table summarizing the association of miRNAs with NEPC

miRNA	Role in	Target	Expression	Altered function	Deregulation	Ref.
miR-663	NEPC (directly through expression of NE markers)	Chromogranin A (CHGA), synaptophysin (SYP), NSE, DHRS7, NKX3.1, DHCR24, PSMA7	Upregulated	Induces NED in cell lines	Human PCa LNCaP cell lines, PC3, Du145	[62,141]
miR-106b, miR-93 and miR-25 cluster	NEPC (directly)	RE-1 silencing transcription factor (REST), p21/WAF1, caspase-7	Upregulated	Induced by hypoxia to cause NED, disease progression and recurrence	Human PCa LNCaP and PC3 cell lines	[65-67]
miR-221-5p	NEPC (directly)	SOCS1, DVL2 (dishevelled 2), p27/Kip1, Ecm29	Upregulated/ Downregulated	Regulates proliferation and migration, induces NED in LNCaP cells, plays tumor suppressive roles	Primary PCa tumor tissue	[68,73]
miR-652	NEPC (directly)	PPP2R3A	Upregulated	Increases aggressiveness in prostate cancer, NED, EMT induction in PC3 prostate cancer cells	PC3 and LNCaP cell lines and PCa xenograft	[74]
miR-301a	NEPC (directly)	β-Catenin and E-cadherin, AR, RUNX3	Upregulated	Regulates epithelial to mesenchymal transition (EMT) in LNCaP cells, activation of Rho signaling in DU145 cells, PCa development and metastasis	LNCaP-AR, LNCaP-AR-ENZR and NCI-H660 cells	[76-79]
miR-708	NEPC (directly)	Sestrin-3, EZH2, CD44, AKT2	Downregulated	Involved in NE differentiation, apoptosis of prostate adenocarcinoma cells	Xenograft human prostate tumors LAPC-4 and LAPC-9, PC3 and LNCaP cell lines	[80,81]
miR-125b	NEPC (indirectly through androgen independence)	BAK1, PUMA/BBC3	Upregulated	Prostatic tumorigenesis and androgen-independent growth	LNCaP cell line	[55]
miR-15a-miR-16-1	NEPC (indirectly through chemoresistance)	CCND1 (encoding cyclin D1) and WNT3A, Bcl 2, IHH	Downregulated	Tumor suppressors, cell survival control, regulate proliferation and invasion	PCa xenograft tumor, PC3 cell line	[16,84,86]
miR-200 family	NEPC (indirectly through chemoresistance)	E-cadherin, ZEB1, ZEB2 JAGGED	Downregulated	Inhibits migration and invasion by epithelial-mesenchymal transition	Metastatic prostate tissue, LNCaP cell line, Du145 cell line, PC3 cell line	[84,89,90,93,94]
miR-320	CRPC	β-Catenin, LAMP 1, LEF-1, CD44, SOX9, Oct-4 and CCND1	Downregulated	Tumor suppressor, decreases PCa tumorigenesis	Primary tumors, PCa cell lines PC3, Du145, LNCaP	[95,101]
miR-31, miR-205	NEPC (indirectly through androgen independence)	E2F6, Bcl-w, HRAS, KLK2 N-chimaerin, ErbB3, E2F1, E2F5, ZEB2, protein kinase C epsilon	Downregulated	Promotes chemotherapeutic agent-induced apoptosis, EMT, inhibits proliferation	LNCaP, PC3 and Du145 cell lines	[102-104,106,107,142]
miR-21	NEPC (indirectly through androgen independence)	PDCD4, TPM1, RECK, MARCKS	Upregulated	Suppresses a network of tumor suppressive pathways	DU145, PC3 and LNCaP cell lines	[109-111]
miR-23b/27b miR34a	CRPC	SIRT1, Bcl 2, CD44, Rac1, cMyc	Downregulated	Inhibits proliferation, survival and metastasis	LNCaP-R1, ALVA31, and PC3 cell lines, Du145 cell line	[27,115,117,118,120,123]
miR-106a-363 cluster	NEPC (directly)	Aurora Kinase A, N-Myc, E2F1 and STAT3	Downregulated	Tumor suppressor	Enzalutamide-resistant LNCaP-AR cells, PDX tumors	[77]
miR-375	NEPC (directly)	ENO2, SYP and CHGA	Upregulated	Oncogenic role	LNCaP-AR, LNCaP-AR-ENZR and NCI-H660 cells	[77]
miR-146a	CRPC	ROCK1, EGFR, MMP2	Downregulated	Tumor suppressive role, inhibits metastasis	PC3 cell line	[124,125]

Listed are the targets that the miRNAs have been shown to directly repress/alter, observed alteration in the miRNA and the corresponding references. miRNA: microRNA; NEPC: neuroendocrine prostate cancer; NED: neuroendocrine differentiation; PCa: prostate cancer; NE: neuroendocrine; AR: androgen receptor; ROCK1: rho-associated, coiled-coil containing protein kinase 1

### miR-663

Following a microarray analysis conducted in 127 PCa patients (CRPC and androgen-dependent prostate cancer), expression of miR-663 increased with Gleason score and clinical stage^[62]^. miR-663 expression was studied in androgen-dependent LNCaP cells, castration-therapy resistant subline LNCaP-androgen-independent, castration-therapy resistant DU145, neuroendocrine-like cell line PC3 and nonmalignant prostate epithelial RWPE cells. miR-663 was more highly expressed in PC3 and DU145 cells than in androgen-dependent cells (LNCaP). Interestingly, conversion of androgen-dependent LNCaP cells to castration-therapy resistant LNCaP-AI led to miR-663 overexpression, suggesting that miR-663 may be involved in the transformation from an androgen-dependent to castration-resistant cell type and may also regulate androgen signaling^[62]^. In addition, miR-663 upregulation led to an increase in the expression of NE markers such as SYP, CHGA and ENO2. miR-663 acts by directly targeting dehydrogenase/reductase SDR-family member 7 (DHRS7), NKX3.1, 24-Dehydrocholesterol reductase (DHCR24) and proteasome 20S subunit alpha 7 (PSMA7)^[62]^. The cited study suggests that miR-663 could serve as a prognostic marker and therapeutic target for CRPC. DHCR24 and DHRS7 may play roles in maintaining the synthesis and/or metabolism of androgens *de novo* in CRPC, gradually contributing to AR reactivation, enhancing PCa progression even though testicular androgen development is curtailed^[63,64]^. Altered expression of PSMA7 can enhance AR transcriptional activity in CRPC and contribute to hormonal progression of PCa^[64]^. Downregulation of NKX3.1 promotes cellular proliferation in CRPC^[64]^.

### miR-93, miR-106b and miR-25 cluster

Hypoxia treatment of neural crest (NC) cell cultures and PCa cell line led to an upregulation of all the miRNAs of the miR-106b~25 cluster (miR-106b, miR-93 and miR-25)^[65]^. The authors proposed that neuronal and neuroendocrine differentiation in NC cells and PCa cells were promoted by hypoxia^[65]^. They suggested that this miR-106b~25 cluster targets and causes a downregulation of the transcriptional repressor RE1-silencing transcription factor (REST), which represses miRNA genes and also neuron-specific protein-coding genes. E2F1and p21/WAF1 were identified as targets of miR-106b, which suppressed the expression of these proteins in LNCaP and PC3 human PCa cells^[66]^. miR-106b-25 cluster directly targets caspase-7 and phosphatase and tensin homolog (PTEN), contributing to cell proliferation, PCa development and disease recurrence^[67]^.

### miR-221-5p

From a comprehensive dataset (GSE21036), expression levels of miR-221-5p were analyzed in 218 PCa patients, and it was reported that miR221-5p targets myriad pathways and could act as tumor suppressor or oncomir depending on experimental and cellular conditions^[68,69]^. miR-221-5p is downregulated during PCa progression, suggesting that miR-221-5p acts as a tumor suppressor in PCa patients^[68]^. miR-221-3p can function as an oncomir or tumor suppressor. Its oncogenic role leads to an increase in cell proliferation by targeting and downregulating p27/Kip1^[68,70,71]^. miR-221-3p can act as tumor suppressor by targeting Ecm29, an oncogene responsible for metastasis, which was observed to be overexpressed in some CRPC specimens^[72]^. A differential expression analyses showed that miR-221-3p acts as an oncomir that contributes to NE differentiation by targeting dishevelled 2, which may underlie androgen-independent PCa^[70]^. To further confirm the oncogenic role, an upregulation of miR 221-5p directly targeted suppressor of cytokine signaling 1 which functions in regulating the MAPK/ERK signaling pathway and epithelial-mesenchymal transition (EMT) features in PCa cells^[73]^.

### miR-652

Five miRNAs (miRs-301a, 652, 454, 223 and 139) were selected from 33 miRNAs after next-generation miRNA sequencing was performed and were identified to be associated with PCa metastasis^[74]^. The authors selected and functionally characterized miR-652 due to its greater potential to be used as a diagnostic marker and reported that miR-652 plays an oncogenic role by directly targeting the “B” regulatory subunit PPP2R3A of the tumor suppressor serine/threonine protein phosphatase 2A (PP2A) thus, leading to its increased ability to promote PCa aggressiveness and NED. PP2A acts as an antiapoptotic protein and influences signaling pathways such as the MAP/ERK family of kinases pathway promoting cell growth, proliferation and survival^[75]^.

### miR-301a

Differential expression analysis of miR-301a was observed in PCa samples on the basis of Gleason score and an upregulation of miR-301a in PCa patients with Gleason 6 and Gleason 7 tumors in comparison to benign prostatic hyperplasia (BPH)^[76]^. The cited authors reported that miR-301a does not only promote cell viability but also activates EMT by regulating the expression of β-catenin and E-cadherin, suggesting that miR-301a is a key driver for enhancing metastatic events in PCa through EMT. Our laboratory recently reported that overexpression of miR-301a leads to induction of NE states coupled with a repression of AR^[77]^. Upregulated miR-301a was strongly associated with increased Gleason score which inhibits the expression of runt-related transcription factor 3 (RUNX3) indicative of its oncogenic role^[78]^. Altered RUNX3 expression in DU145 cells activates Rho signaling which contributes to PCa development and metastasis^[79]^.

### miR-708

miR-708 and miR-378c were found to be key miRNAs; after miRNA profiling expression was conducted in luminal PCa cells, their corresponding NEPC cells showed that miR-708 was reduced significantly in NEPC tumors as compared with miR-378c. miR-708 acts by directly targeting sestrin-3^[80]^. The sestrin-3 gene has been identified as a contributing factor in the increase of intracellular reactive oxygen species^[80]^. In addition, the reduction of miR-708 activity contributes to the initiation, progression and growth of prostate cancer by controlling the function of both CD44 and AKT2^[81]^. CD44, a cell adhesion molecule, has been identified as a prevalent marker of PCa^[82]^. Overexpression of EZH2, a target of miR-708 increased expression of NE markers CHGA and CHGB in PC3 and LNCaP cells^[61]^. EZH2, which is regulated by cyclin-dependent Kinase 1 and Wnt signaling, may also be a contributing factor in the transdifferentiation of PCa cells to NEPC^[80]^.

### miR-125b

miRNA-125b has been shown to be involved in the development of castration resistance^[55]^. Using microarray analysis, the authors analyzed differential expression of 132 miRNAs in androgen-dependent (AD) LNCaP cells and its related androgen-independent (AI) subline^[55]^. Their data showed that AI LNCaP cells expressed 5-fold increased levels of miR-125b when compared with the androgen-dependent parental LNCaP cells. This suggests that miRNA-125b may contribute to castration resistance and further progression to NEPC by enhancing androgen independent growth. Furthermore, miR-125b directly targets Bcl2 antagonist/killer 1 (Bak 1) by reducing its expression, thus disrupting mitochondrial apoptosis pathway. miR-125b also targets pro-apoptotic signaling mediators PUMA/BBC3 that displace the inhibitory effect of Bcl2 on Bak1. Confirmatory studies in AD and AI PCa cells have therefore reported that the androgen signaling pathway is able to regulate miR-125b expression, which leads to downregulation of Bak1, hence underscoring the oncogenic effect of miR-125b in stimulating androgen-independent growth of PCa cells^[83]^.

### miR 15a and miR-16-1

miR-15a/miR-16-1 is a cluster that is located at chromosome 13q14, a region which has been shown to be frequently lost in advanced to metastatic PCa, thereby causing downregulation of the cluster^[84]^. Studies in PCa xenograft tumor *in vivo* detailed that downregulation of mir-15a/miR-16-1 enhances cell survival, proliferation and invasive properties. Loss of miR-15/miR-16 plays a synergistic effect along with upregulated miR-21 in increasing aggressiveness and acquisition of a strong EMT phenotype in PCa metastasis^[85]^. miR-15a/miR-16-1 directly targets B-cell lymphoma 2 (Bcl2), cyclin D1 (CCND1) and wingless-type 3A (WNT3A) in advanced PCa^[84]^. Indian Hedgehog (IHH) was also found to be a direct target of miR-15a/miR-16-1 cluster by its significant upregulation in cell lines derived from metastatic tumors, specifically the PC3 cell line^[85]^. IHH promotes the proliferation of tumors through direct transcriptional control of the polycomb gene Bmi-1, which has been implicated in PCa development and progression^[85]^. WNT3A signaling increases the expression of β-catenin and activates survival and proliferation pathways through the phosphorylation of ERK and Akt^[86]^. Bcl2, an antiapoptotic protein contributes to PCa progression by enhancing the differentiation of LNCaP PCa cells from an androgen-dependent to an androgen-independent cell type^[87]^. Cyclin D1 is a positive regulator of cell cycle (G1 phase) that plays a crucial role in tumorigenesis^[88]^. Furthermore, downregulation of miR-15/miR-16 cluster decreases the sensitivity of PCa cells to docetaxel, one of the standard drugs for androgen-independent prostate tumors^[86]^.

### miR-200 family

The miR-200 family, consisting of miR-141, miR-200a, miR-200b, miR-200c and miR-449, is reported to be downregulated during PCa progression; these miRNAs suppress PCa tumor metastasis through the inhibition of EMT, which contributes to chemoresistance^[84,89]^. Low expression levels of ZEB1 and vimentin and a high expression of E-cadherin were reported in the Du145 cell line, upon overexpression of miR-200c suggesting that miR-200c can inhibit PCa cells from proliferating and undergoing EMT, leading to inhibition of invasion and migration^[90]^. Transcription factor ZEB1 regulates gene expression by binding to ZEB-type E-boxes (CACCTG) within the promoter region of the target genes to silence the genes promoting cell migration and tumor metastasis^[91,92]^. ZEB2 is known to downregulate myriad genes that code for crucial proteins responsible for the epithelial phenotype, including E-cadherin^[92]^. In addition, miR-200c directly targets JAGGED1, curtailing proliferation of human metastatic PCa cells (PC3 cells)^[93]^. A high level of JAGGED1 shows a positive correlation with PCa recurrence and aggressiveness^[94]^.

### miR-320

miR-320 overexpression in PCa cells plays a tumor-suppressive role by decreasing PCa tumorigenesis *in vitro* and *in vivo* through targeting of lymphoid enhancer-binding factor 1 (LEF1), CD44, SOX9, Oct-4 and CCND1, all associated with the Wnt/β-catenin signaling pathway^[95]^. LEF1 has been indicated to regulate the expression of AR and is overexpressed in androgen-independent PCa^[96,97]^. CCND1 plays a role in the resistance of CRPC patients to ENZ treatment^[98]^. Sox9 enhances the development of PCa to therapy resistance by influencing the activity of the NF-kB pathway^[99]^. Elevated increase in POU transcription factor OCT4 in CRPC patients after docetaxel chemotherapy, suggests its role in tumorigenesis and aggressiveness of CRPC^[100]^. In addition, miR-320a directly targets lysosomal associated membrane protein 1, which is overexpressed in PC3 and Du145 cells and CRPC clinical specimens^[101]^.

### miR-205 and miR-31

miR-205 and miR-31 are significantly downregulated in WPE1-NB26 cells, LNCaP, Du145 and in other advanced-stage PCa cell lines^[102]^. Downregulation of these two miRNAs target antiapoptotic genes BCL2L2 (encoding Bcl-w) and E2F6 which are shown to be highly expressed in Du145, LNCaP and PC3 cell lines^[102,103]^. Overexpression of miR-205 in PCa cell lines reduced cell invasion by downregulating protein kinase Cε (PKCε)^[104]^. PKCε, a serine/threonine kinase member of the PKC subfamily plays a role in sustaining migration and invasion in PCa with its expression correlating with aggressiveness of the disease by transforming androgen-dependent LNCaP cells into an androgen-independent variant^[105]^. In addition, miR-205 has been shown to directly target ZEB2, N-chimaerin, ErbB3, E2F1 and E2F5 in PCa cells^[104]^. miR-205 represses ZEB2, resulting in an upregulation of E-cadherin to acquire an epithelial-like phenotype^[104]^. miR-205 downregulation of E2F5, E2F1 and ErbB3, a member of the epidermal growth factor receptor family, could also halt PCa progression and invasive ability^[104]^. miR-205 may act synergistically with miR-130a and miR-203 to repress AR and MAPK, thereby interfering with processes involved in castration resistance^[106]^. Other plausible targets of miR-205 are KLK2 in the human kallikrein gene family and HRAS^[106,107]^.

### miR-21

miR-21 plays an oncogenic role in PCa, and an increase in expression of miR-21 has been shown to correlate with cancer recurrence in PCa patients following radical prostatectomy^[89]^. Overexpression of miR-21 in PC-3 and DU145 cell lines has been shown to contribute to AR-independent PCa growth and also to induce castration resistance phenotype^[108]^. miR-21 directly targets myristoylated alanine-rich protein kinase c substrate (MARCKS) which plays a crucial role in cell motility and invasion in DU145, PC3 and LNCaP cell lines^[109]^. miR-21 also directly targets the matrix metalloproteinase (MMP) inhibitor reversion-inducing cysteine-rich protein with Kazal (RECK) motifs, a key inhibitor of several MMPs, thereby enhancing cell invasion in the Du145 cell line^[110]^. miR-21 has also been studied and proven to partly regulate programmed cell death protein 4 (PDCD4) and tropomyosin 1 (TPM1)^[109]^. A gain-of-function study conducted on miR-21 showed that elevated levels of miR-21 in an androgen-dependent PCa cell line was enough to drive androgen-independent growth^[108]^. Elevated levels of miR-21 in mouse models were found to enhance tumor growth and an induction of a castration-resistant phenotype^[108]^. Functional studies confirmed that an increase in resistance to docetaxel (chemotherapy for CRPC) in PC3 wild-type cells was as a result of overexpression of miR-21 which directly targeted PDCD4^[111,112]^.

### miR-23b/27b and miR-34a

miR-23b and miR-27b cluster is found on human chromosome 9 and has been reported to be downregulated in CRPC tumors^[113,114]^. miR-23b/27b expression was shown to decrease invasiveness in two independent aggressive CRPC cell lines, ALVA31 and PC3-ML^[115]^. Downregulation of miR-23b/27b results in decrease in expression of E-cadherin and the increase in Rho GTPase Rac1 activity which plays a key role in aggressive PCa, specifically in the PC3 cell line, through regulation of cytoskeleton rearrangement essential for cell migration^[115,116]^. The increase in Rac1 activity was also confirmed in the androgen-independent cell lines LNCaP-R1, ALVA31 and PC3 cells compared to the androgen-dependent LNCaP cells^[117]^. Tumor suppressor miR-34a which has been shown to be downregulated in CRPC exerts its action by directly targeting c-myc to inhibit cell migration and invasion particularly in PC3 cell line^[118,119]^. Downregulation of miR-34a has a direct effect on SIRT1 and Bcl2 which leads to the development of paclitaxel resistance in hormone-refractory PCa which causes metastasis and death in CRPC patients^[120]^. In addition, CD44, a cancer stem cell marker has been shown to be selectively expressed in NEPCs^[121]^. A study reported nearly 100% positive expression of CD44 in PC3 cells and approximately 60% in DU145 cells^[122]^. miR-34a enhances antitumor and antimetastatic properties in tumorigenic CD44+ PCa cells by directly targeting CD44^[123]^.

### miR 106a-363 cluster

Studies conducted in our laboratory using patient samples with CRPC-adeno (CRPC patients with adenocarcinoma features) and CRPC-NE tissues (CRPC patients with NE characteristics) showed that the miR-106a~363 cluster (miR-106a, miR-20b and miR-363) is downregulated in CRPC-NE tissues^[77]^. Expression analyses in PCa cell lines (PC3, LNCaP) compared to BPH1 showed that miR-106a~363 cluster expression was decreased and significantly downregulated in ENZ-resistant LNCaP-AR cells as compared to corresponding parental LNCaP-AR cells, suggesting a correlation between the downregulation of these miRNAs with ENZ resistance. We found that these miRNAs drive NEPC by targeting AURKA, MYCN, E2F1 and STAT3.

### miR-375

Our laboratory performed experiments on patient samples with CRPC-adeno and CRPC-NE tissues and reported that miR-375 was upregulated in CRPC-NE tissue samples^[77]^. Expression analyses in PCa cell lines (PC3 and LNCaP-AR, LNCaP-AR-ENZR and NCI-H660) showed a significant increase in expression of miR-375 suggesting its role in NEPC. We further found an induction of NE genes ENO2, SYP and CHGA by miR-375 overexpression^[77]^.

### miR-146a

Tumor suppressor miR-146a has been shown to be downregulated in CRPC tissues; miR-146a downregulation inhibited the growth and migratory ability of androgen-independent human CRPC cell lines PC3 and Du145, which may contribute to CRPC progression in these cell lines^[124]^. miR-146a directly targets EGFR through the EGFR/ERK signaling pathways, which enhances proliferation and metastasis in CRPC^[124]^. Another target for miR-146a was shown to be MMP2, a gene known to promote tumor progression^[124]^. Another study showing the effect of downregulation of miR-146a in androgen-independent PC3 cells reported that increase in expression of Rho-associated, coiled-coil containing protein kinase 1 (ROCK1) enhances cell proliferation, invasion and metastasis by activating the PI3K-mediated Akt/TOR/eIF4E signaling pathway^[125]^.

## miRNAs as biomarkers for detection and prognosis and therapeutic targets in NEPC

A significant number of studies suggest an important role of miRNAs as biomarkers for detection and prognosis in the management of PCa, especially the NE variant^[126,127]^. Some of these miRNAs have also been shown to be a novel therapeutic target in the treatment of advanced forms of PCa. miR-15a and miR-16 which act as tumor suppressors by targeting CCND1, WNT3A and Bcl2 may be considered to have therapeutic potential in NEPC by either acting independently or being used as a combinational therapy with chemotherapy^[86]^. miR-146a, shown to play a tumor suppressive role in androgen-independent prostate cancer, have been postulated as a promising therapeutic target by directly targeting ROCK/caspase 3 pathway^[128]^. miR-573, which targets FGFR1 by modulating EMT and metastasis of PCa cells, may also serve as a therapeutic target or potential biomarker in managing PCa^[129]^. miR-21, which has been shown to be an oncomir and plays a critical role in suppressing a network of tumor suppressive pathways by targeting PDCD4 may serve as a therapeutic target or biomarker in PCa^[130]^. Overexpression of miR-106b, miR-93 and miR-25 cluster may contribute as a clinical biomarker in advanced PCa by targeting transcriptional repressor REST^[65]^. miR-708 could serve as a prognostic marker by targeting EZH2, which binds to the miR-708 promoter and silences it in NEPC^[80]^. miR-652, an oncomir targeting PPP2R3A could function as a biomarker and a therapeutic target for aggressive prostate cancer, considering its contribution to tumor progression by promoting NED^[74]^. Increased levels of miR-141-3p and miR-375-3p found in plasma may serve as a prognostic marker in predicting progression in mCRPC patients treated with ABI or docetaxel^[131]^. Five miRNAs, miRs-301a, -652, -454, -223 and -139, which have an association with metastasis in PCa, could serve as novel prognostic markers in the management of PCa^[132]^. miR-301a, which plays an oncogenic role in PCa by targeting AR, could be a potential marker for metastatic conditions in PCa patients^[77]^.

## Pros and cons of miRNAs in therapeutics

Dysregulation of miRNA expression has been implicated in several cancers^[133]^. Treatments based on miRNAs have been fully explored and due to their ability to regulate a wide array of genes by targeting many mRNAs, they can be effective in the control of cancers, thereby having some added advantage over conventional drugs^[134]^. In addition, it is easy to design these miRNA such as miRNA mimics, and these miRNA-based drugs can also be highly specific and potent^[135,136]^. Another advantage of miRNA-based drugs is their small molecular size and low cost of synthesis^[134]^. A major drawback in miRNA-based therapy is with drug delivery; nucleases in serum and cells of the immune system degrade these exposed miRNAs when systematically injected^[134,135,137]^. In addition, due to the fact that miRNAs can target myriad genes, there is a problem of off-target effects which can be lethal to non-targeted cells^[138-140]^.

## Conclusions and perspectives

Therapy-induced NEPC, which occurs following second-line androgen pathway inhibitor treatment is very aggressive and is associated with poor survival rates. The mechanism for this NED is not yet completely understood. miRNAs have been studied and have been shown to play significant roles in regulating PCa cell growth, apoptosis, invasion and migration abilities. Experiments conducted as discussed in this review, so far have shown a strong correlation of miRNAs with advanced PCa progression. Recent research has also shown that some of these miRNAs can play significant roles in NEPC by acting as oncomirs and tumor suppressors making them very relevant prognostic markers and therapeutic targets in the management of NEPC. However, more miRNA profiling studies need to be conducted to ascertain the etiology of NEPCs since they have not been extensively studied in the field. Also, the functional roles of miRNAs need to be studied in more detail. Eventually, the goal is to translate miRNA research to clinical settings. This field has not yet been investigated and most of the studies have so far focused on preclinical studies on miRNAs.

## Declarations

### Copyright

© The Author(s) 2020.
